# A Comprehensive Review on the Efficacy of Nerve Blocks in Reducing Postoperative Anesthetic and Analgesic Requirements

**DOI:** 10.7759/cureus.38552

**Published:** 2023-05-04

**Authors:** Jason Jogie, Joshua A Jogie

**Affiliations:** 1 Anesthesiology, Port of Spain General Hospital, Port of Spain, TTO; 2 Medicine, University of the West Indies, St. Augustine, TTO

**Keywords:** local anesthetics, analgesic consumption, anesthetic requirements, postoperative pain management, nerve blocks

## Abstract

The purpose of this article review is to investigate whether or not nerve blocks are beneficial in minimizing the amount of postoperative anesthetic and analgesic medication required, hence improving patient outcomes and reducing healthcare costs. This review investigates several different kinds of nerve blocks, their administration techniques, and the anatomical and physiological aspects that influence nerve block effectiveness. It analyzes the impact of nerve blocks on opioid use, postoperative pain scores, and the incidence of opioid-related adverse effects by compiling the findings of numerous large-scale, randomized, controlled trials.

Infection, hematoma, nerve injury, and systemic toxicity are some potential complications of nerve blocks discussed in the article. It concludes with recommendations for optimizing nerve block techniques in clinical practice and identifies areas that require further research, such as the development of new anesthetics and the identification of patient subgroups that would benefit the most from nerve blocks. In addition, it provides recommendations for optimizing nerve block techniques in clinical practice.

## Introduction and background

Postoperative pain control is a crucial component of patient care since it can have a detrimental influence on patients' recovery, satisfaction, and use of hospital resources [1]. Opioids have traditionally served as the foundation for postoperative pain management; however, their use is associated with various adverse side effects. These include the risk of addiction, respiratory depression, constipation, nausea, vomiting, drowsiness, and impaired cognitive function [2]. As a result, there has been an increase in interest in researching alternative pain management methods, such as nerve blocks and regional anesthetic techniques [3]. Nerve blocks deliver tailored analgesia with fewer systemic adverse effects by administering local anesthetics to certain nerves or nerve plexuses [4]. Based on a synthesis of pertinent research articles and clinical trials, this article review seeks to analyze the effectiveness of nerve blocks in reducing postoperative anesthetic and analgesic requirements.

Nerve block types

Peripheral, central, and neuraxial nerve blocks can all be generically categorized as having different indications, anatomical considerations, and technical considerations [5]. Peripheral nerve blocks (PNBs), which are frequently used during procedures on the extremities [6], entail administering local anesthetics to specific peripheral nerves, such as the femoral, sciatic, or brachial plexuses. Central nerve blocks are used for various surgical procedures, including those on the abdomen, the thorax, and the lower extremities [7]. They target the spinal cord or surrounding structures, such as the epidural or subarachnoid area. For procedures involving the lower abdomen, pelvis, and lower extremities, neuraxial blocks, such as spinal and epidural anesthesia, entail the administration of local anesthetics into the subarachnoid or epidural area, respectively [8].

Physiological and anatomical considerations

The accuracy of the pertinent anatomy, including the target nerves' courses and branching patterns, as well as the surrounding structures, including blood vessels and fascial planes, is crucial for the success of nerve blocks [9]. The practice of regional anesthesia has been transformed by ultrasonography guidance, which enables real-time viewing of the target nerves and surrounding tissues and makes it easier to administer local anesthetics precisely [10]. In addition to ultrasound, electrical nerve stimulation and nerve stimulators are further methods for localizing nerves [11]. The effectiveness and safety of nerve blocks can also be affected by patient-specific factors, including body habitus, anatomical differences, and coexisting diseases [12].

The actions of local anesthetics on nerve fibers, which prevent the production and propagation of action potentials, are the primary mediators of the analgesic effects of nerve blocks [13]. Using voltage-gated sodium channels, local anesthetics block the entry of sodium ions and the subsequent depolarization of the neuronal membrane, which is how they work [14]. The chemical structure, lipid solubility, protein binding, and pKa of local anesthetics, as well as their onset, duration, and potency, are all influenced by these variables [15].

Effectiveness of nerve blocks

The effectiveness of nerve blocks in lowering postoperative anesthetic and analgesic requirements has been the subject of numerous scientific studies and clinical trials [16-20]. Compared to patients receiving systemic analgesia or general anesthesia alone, most of these studies have found that patients receiving nerve blocks experience much lower levels of opioid consumption, pain scores, and opioid-related adverse effects [21-23]. Furthermore, certain research [24-26] has shown the potential advantages of nerve blocks in terms of shorter hospital stays, higher patient satisfaction, and faster functional recovery.

Potential complications and limitations

Despite having many benefits, nerve blocks have possible drawbacks and restrictions in clinical practice [27]. Infection, hematoma, nerve damage, and systemic toxicity are a few examples of potential complications [28]. Although the risk of these is usually low, if they do occur, there could be severe morbidity and unfavorable patient outcomes [29]. Inadequate anesthetic, technical difficulties, and patient-related issues such as anatomical variations and concomitant diseases are potential causes or complications of nerve block failure [30,31]. Additionally, the unavailability of commercial block needles and ultrasound or nerve stimulators presents challenges for adopting peripheral nerve blocks in low-resource settings. However, some authors have reported success using improvised methods, such as cannula-stylets, instead of costly commercial block needles [32]. This innovation demonstrates the potential for effective peripheral nerve block practices even in resource-limited environments.

Clinical practice recommendations and future research directions

Based on the data from the trials that have been examined, nerve blocks appear to be a viable strategy for lowering the need for postoperative anesthesia and pain medication, with potential advantages for patient outcomes and healthcare expenditures [33,34]. Using nerve blocks may be most beneficial for some patient subgroups, such as those with a history of opioid abuse, patients at high risk for postoperative complications, and individuals with severe chronic pain. However, more research is warranted in view of developing innovative anesthetic drugs, enhancing nerve block procedures, and accurately identifying these patient subgroups [35,36]. Further investigation into these subpopulations' specific needs and characteristics will enable clinicians to provide more targeted and effective pain management strategies. Moreover, evidence-based recommendations, relative advantages, potential complications of nerve blocks, and case-based specific requirements should be followed and noted when implementing them in clinical practice [37].

## Review

Types of nerve blocks and anatomical considerations

For a wide range of surgical operations, peripheral nerve blocks (PNBs) have been utilized to deliver targeted anesthetic and analgesia. These blocks are especially helpful when general anesthesia may not be an option or when minimal sedation is preferred. By reducing the systemic opioid requirement and its associated complications, PNBs can enhance patient outcomes [38].

Single-shot PNBs do not require the insertion of a catheter for the continuous injection of local anesthetics [39]. Among the most popular PNBs are the femoral nerve block, brachial plexus block, and lumbar plexus block [40-42]. Each block targets a particular anatomical region and can effectively reduce surgical site pain. Femoral nerve blocks, for instance, can be utilized for knee surgery, while brachial plexus blocks and lumbar plexus blocks can be used for upper extremity and lower extremity surgeries, respectively.

The efficacy and safety of PNBs have improved with ultrasonography [43]. Ultrasound guidance allows for an improved success rate for PNBs and a decreased risk of complications. It also allows for real-time imaging of the needle and the targeted nerves. Owing to their ease of use and improved outcomes, ultrasound-guided PNBs have been the procedure of choice for many medical professionals [38].

Nerve damage, hematoma formation, and infection are notable complications of PNBs [38]. Nerve injuries can be temporary or permanent, with rates varying significantly in the literature. Temporary nerve injuries have been reported to occur in 3-15% of cases, while permanent nerve damage is relatively rare, with an incidence of 0.03-0.4% [44]. Hematoma formation and infection rates are also relatively low, ranging from 0.4-4.2% and 0.02-2.7%, respectively [45]. Understanding these risks can help clinicians make informed decisions when selecting appropriate pain management techniques for their patients. 

Physiological basis and pharmacology

Peripheral nerve blocks (PNBs) work by preventing the propagation of nerve impulses along a nerve by blocking ion channels with local anesthetics [46]. Local anesthetics specifically limit the propagation of action potentials and cause a loss of sensory and motor function in the targeted area by blocking voltage-gated sodium channels in nerve fibers [47]. Many variables, such as the class of local anesthetic agent employed, the concentration, volume, and total dose of the agent injected, and the nerve or plexus targeted, affect the extent and duration of nerve blockade [47].

Adjuvants, such as clonidine, sodium bicarbonate, and ketamine, have been added to local anesthetics in peripheral nerve blocks (PNBs) to extend the analgesic duration and enhance nerve blockade quality without causing adverse effects. Dexmedetomidine, a selective alpha-2 agonist, and dexamethasone, a glucocorticoid, both exhibit comparable efficacy in prolonging analgesia and improving PNB outcomes, including postoperative pain, when combined with local anesthetics [48-49].

Another strategy for improving pain management in individuals having surgical operations is multimodal analgesia. This method uses analgesic modalities to target various parts of the pain pathway, providing improved pain relief with fewer side effects [50]. Together with other analgesics like opioids and nonsteroidal anti-inflammatory medications (NSAIDs), PNBs can be utilized as a part of multimodal analgesia [50]. Opioids are strong analgesics that function by utilizing opioid receptors in peripheral tissues and the central nervous system. They can be used in conjunction with PNBs for improved analgesia [50]. Opioid use, however, is linked to several negative side effects, including respiratory depression, drowsiness, and nausea [50]. NSAIDs are an additional class of analgesics that can be taken in conjunction with PNBs. They function by preventing the synthesis of prostaglandins, which mediate pain and inflammation [50]. However, NSAIDs are linked to complications, including renal dysfunction and gastrointestinal bleeding [50].

Efficacy in reducing postoperative anesthetic and analgesic requirements

For various surgical procedures, peripheral nerve blocks (PNBs) have been demonstrated to offer greater pain management while lowering the amount of postoperative analgesic medicine needed. PNBs are an important tool for clinicians to successfully treat postoperative pain and reduce the chance of opioid-related side effects [51-53].

For instance, compared to systemic analgesics alone, PNBs have been reported to give greater analgesia after shoulder surgery. Concerning shoulder-related procedures, it has been demonstrated that a single-shot interscalene block is extremely successful at relieving pain [54]. Similar results have been observed for continuous femoral nerve blocks, which have been shown to decrease the need for opioids after total knee arthroplasty [55].

PNBs have also been linked to a decreased incidence of postoperative complications and opioid-related adverse events, in addition to improved pain management. The use of PNBs was linked to decreased incidence of complications in individuals who had undergone hip and knee arthroplasty, according to a population-based study [56]. PNBs have also been demonstrated to lower the frequency of opioid-related side effects like respiratory depression, nausea, and vomiting [51,52].

Continuous peripheral nerve blocks (CPNBs), such as continuous femoral nerve block or sciatic nerve block, have been demonstrated to be more effective at controlling pain than systemic opioids, according to a meta-analysis that compared the effectiveness of both treatment methods [57]. These CPNBs involve the continuous infusion of local anesthetics through a catheter placed near the target nerve, providing sustained analgesia for an extended duration, which can vary depending on the specific nerve block and patient's needs. Although this study highlights the superiority of CPNBs in pain management, it does not explicitly address the cost-effectiveness of the technique or complications such as infection. Further investigation into these aspects would provide a more comprehensive understanding of the overall benefits and potential drawbacks of continuous PNBs. Continuous PNBs offer long-lasting pain relief and may reduce the likelihood of unfavorable opioid-related events linked to systemic opioid use [57].

Potential complications and limitations

Although peripheral nerve blocks (PNBs) provide numerous advantages for treating pain, doctors must be aware of also possible risks and limitations. Although uncommon, neurological problems such as nerve injury following PNBs might have substantial repercussions for patients [58]. Furthermore, possible CPNB complications related to catheter use include infection and bleeding [59].

Local anesthetic systemic toxicity (LAST), a rare but potentially fatal side effect of local anesthetics, is another potential issue related to PNBs. However, it has been demonstrated that the risk of LAST is reduced when PNBs are performed under ultrasound guidance [60]. Ultrasound guidance improves the precision of needle insertion, reducing the need for more local anesthetics and lowering the danger of systemic toxicity. On the other hand, ultrasound-guided PNBs have a challenging learning curve. To perform ultrasound-guided nerve blocks successfully and safely, one needs to receive the necessary initial practice and close supervision, as failing to do so increases the risk of complications [61].

There are certain limitations associated with PNBs in addition to these possible complications. PNBs, for instance, should not be used on patients with certain medical conditions, such as blood disorders, local anesthetic sensitivities, or infections at the injection site [58]. PNBs also call for a certain degree of patient awareness and participation, which may be problematic for some patient populations, like young children or those with cognitive difficulties [59].

Although PNBs provide many advantages for treating pain, there are also potential disadvantages and limitations that medical professionals must consider before using them. PNBs, especially with continuous blocks, have the potential to cause neurological problems, infections, hemorrhages, and issues with catheters. Overall, medical professionals must carefully weigh the advantages and disadvantages of PNBs and decide which approach is best for each patient on an individual basis.

Clinical practice recommendations and future research

Peripheral nerve blocks (PNBs) may be an important part of a multimodal analgesia strategy for various surgical operations. To improve the success rate of the block and lower the risk of complications, ultrasonography guidance should be used as much as possible during PNBs [62]. Clinicians should know the indications, contraindications, and procedures associated with different PNBs to optimize benefits.

Ongoing research is required to enhance the techniques and identify the most efficient local anesthetic and adjuvant combinations for PNB usage. While it has been demonstrated that adjuvants such as dexmedetomidine and dexamethasone extend the pain relief time and quality brought on by PNBs, more research is required to determine their long-term safety and efficacy [63].

More studies are required to comprehend the long-term effects connected to the use of PNBs. PNBs are effective and safe in short-term studies, but longer-term studies are needed to assess their effects on patient outcomes such as quality of life, functional status, and healthcare use. Research in this field will assist in clarifying the function of PNBs in treating postoperative pain and in finding ways to enhance patient care.

## Conclusions

This article review analyzed the effectiveness of utilizing nerve blocks to reduce the need for postoperative anesthetic and analgesic medications. Several surgical procedures have been demonstrated to benefit significantly from nerve blocks, including decreased postoperative pain, reduced need for opioids, and increased patient satisfaction. Moreover, improvements in ultrasound-guided procedures have helped administer nerve blocks with improved accuracy and safety.

However, while applying nerve blocks in clinical practice, various complications and limitations must be considered. They include the potential for systemic toxicity from local anesthetics, nerve damage, and infection. To reduce these risks and improve patient outcomes, it is essential to adhere to best practices and receive the appropriate training.

According to recent research, nerve blocks are a useful part of multimodal analgesia for various surgical operations. However, further research is required to improve and standardize procedures and long-term safety and pinpoint the ideal patient demographics who would benefit the most from nerve blocks. Future research should compare various adjuvants and their combinations to improve nerve block efficacy and security. All things considered, nerve blocks show promise as a crucial technique in the perioperative management of pain. Clinicians can dramatically improve patient outcomes and satisfaction in the postoperative period by using nerve blocks in their approach to pain.
